# Stomatognathic evaluation at five years of age in children born premature and at term

**DOI:** 10.1186/s12887-015-0343-6

**Published:** 2015-03-29

**Authors:** Kíldane Maria Almeida Guedes, Alzira Maria D’Avila Nery Guimarães, Alliny de Souza Bastos, Karoline Guedes Mesquita Salviano, Neuza Josina Sales, Maria Luiza Dória Almeida, Ricardo Queiroz Gurgel

**Affiliations:** Graduate School of Health Sciences, Federal University of Sergipe, Aracaju, Brazil; Department of Nursing, Federal University of Sergipe, Aracaju, Brazil; Araraquara School of Dentistry, Univ. Estadual Paulista, Araraquara, Brazil; Universidade Tiradentes, Aracaju, Brazil; Federal University of Sergipe, Aracaju, Brazil; Center for Postgraduate Medicine, Federal University of Sergipe, Aracaju, Brazil; Core Postgraduate Medicine, Federal University of Sergipe, Aracaju, Brazil; Center of Postgraduate Medicine, PhD Sciences of the Federal University of Sergipe, Aracaju, Brazil

**Keywords:** Dental Enamel Hypoplasia, Dentition, Primary, Prematurity, Palate, Malocclusion

## Abstract

**Background:**

The high frequency of alterations of the stomatognathic system associated with premature birth may suggest that prematurity is an important risk factor in the development of this system. Prematurity has an incidence between 6-11% of births and is associated with factors such as genetic, maternal conditions (obstetric problems, nutritional status, infections) and antenatal care. In addition, undesirable situations, such as changes in enamel and the development of the skeletal structure, also appears to be associated with prematurity. This study aimed to look for changes in the stomatognathic system at five years of age associated with premature birth.

**Methods:**

We estimated the prevalence of developmental disorders of the stomatognathic system in the primary dentition of preschool children at five years of age. Changes in preterm infants (n = 32) compared with term born (n = 381) were evaluated . Clinical examinations and questionnaire with sociodemographic and health of mothers and children information. Gestational age, birth weight, head circumference, Apgar score and mechanical ventilation, were collected from the medical records to birth records. The explanatory variable was preterm (<37 weeks gestational age).

**Results:**

Results: Prevalence of 7.7% of preterm infants was found. Of these, 40.6% had atresic palate, 56.2% malocclusion and 21.8% enamel hypoplasia. Forty (9.6%) children were not breastfed at the breast, and 26 (65.0%) had some type of malocclusion, showing association between not breastfeeding with an abnormal development of the stomatognathic system. The group of preterm infants showed five times more changes in head circumference and three times more mechanical ventilation use at birth. Change in head circumference at birth and mechanical ventilation has a significant association between groups of preterm and term infants.

**Conclusions:**

Mechanical ventilation at birth directly contributed to an increased risk of developmental disorders of the stomatognathic system in preterm infants, especially dental hypoplasia. Non-breastfed children had a higher risk of developing malocclusion. Alterations in head circumference were related effective on dental malocclusion. The results suggest that changes in the stomatognathic system are influenced by premature birth and points to the imperative need of using methods of preventive.

## Background

Premature birth, a major public health challenge in Brazil, occurs in 6-11% of births and is associated with the large majority of infant and childhood deaths. Premature delivery may result in high financial costs and potential social and economic problems for the affected families [1]. The prevention of premature births and good quality aftercare are therefore of utmost importance.

The identification of factors associated with premature birth and the reduction of its harmful consequences is challenging due to the complexity and multi-causal relationships of the variables involved. Associations with genetic factors, obstetric care, nutritional status, infections, toxic exposure, antenatal care, demographic, psychosocial and environmental problems have been reported [2,3]. However, the effects on the enamel of the teeth and development of the skeleton structure, specifically the palate and tooth eruption delayed still need to be explored in the literature.

Previous population-based studies have not adhered to any specific standards; therefore it is difficult to compare results between studies. There is ample evidence that certain chronic diseases and unfavorable characteristics may originate during fetal life, e.g., cardiovascular disease and diabetes [4]. However, the association between preterm birth and palate atresia, malocclusion and dental hypoplasia has been less intensively studied. The early detection of risk factors for the occurrence of these alterations is essential for a better understanding of the problem and may lead to the development of appropriate prevention and control strategies.

Epidemiological studies on oral health in early childhood describe the changes in the stomatognathic system and help to establish preventive and educational programs that may control the symptoms and reduce the costs incurred [5]. This study aims to investigate Stomatognathic system at five years of age associated with premature birth cohort from the region of Aracaju/SE. We hypothesize that factors such as preterm birth, low socio-economic status and lack of breastfeeding are associated with a higher risk of abnormal development of the stomatognathic system.

## Methods

This research is characterized by being a living longitudinal study of a birth cohort, to estimate the prevalence of developmental disorders of the stomatognathic system in the deciduous dentition of 413 five-year-old pre-school children from Aracaju, Sergipe. These children were from a cohort of births registered in the four maternity hospitals in the metropolitan area of Aracaju (ESPHA) in the period from March 8^th^ to July 15^th^ 2005. For a detailed methodology describing aspects of delivery and childbirth see our previous study [6]. The final number of the sample was given depending on the result of the active pursuit of the cohort population.

Initially, individuals were identified during visits to schools in the city of Aracaju, health units and Reference Centers. Specially trained staff interviewed mothers or guardians and completed survey forms containing the child’s overall development and detailing their socio-demographic, socio-economic, nutritional status and any behavioral, speech, hearing or associated diseases. Data collection was blind with regard to the children’s birth condition. The resulting data was divided according to gestational age: in preterm infants up to 36 weeks and six days (n = 32) and term infants, 37 completed weeks or more (n = 381). A paragraph that explicitly says the sample limitations in this research was added in the method. The limitation of sample numbers was mainly due to absence of children on their schools, with frequent medical certificates caused by common diseases. The professors state that these families go through long periods of unemployment or frequent employment changes of one or of all family members. To work around this reality, families seek amendment of the residential lease and the number of fixed or mobile phone and a plan of lower cost, which hampers its location. Another strategy used during the school year is the change of city and state, and often the child goes to live with relatives in other municipalities. This situation causes weakness of family bonding and of the school links, at least transiently. The economic reality of the family, possibly generates absenteeism and dropout of students in various phases of the school year, which possibly hinder the process of teaching and learning. This may also be a reason for the small proportion of the original cohort children that was found.

**Figure 1:**
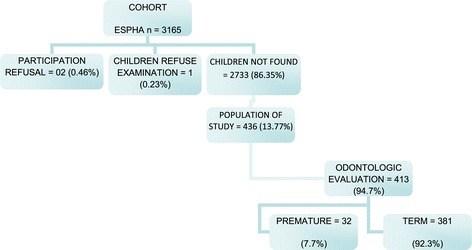
**Study population.**

The final number of children evaluated in this study was significant and became very relevant especially when compared with previous research, and we have larger sample than before [7,8]. The economic reality of the family, possibly generates absenteeism and dropout of students in various phases of the school year that possibly hinder the process of teaching and learning and creating great difficulty in locating (Figure 1).

The tests for the evaluation of the stomatognathic system were conducted using a collection tool designed especially for this survey. Eligible participants were examined between March 2010 and January 2011. These children were assessed for palatal development by measuring intercanine distance, changes of enamel, malocclusion and abnormal number of dental units. Prior calibration was performed between the chief examiner and the examiner’s assistant, in case there was any need for the replacement of the dentist. The validation of the calibration was measured using a Kappa test for standardization of tests resulting in a weighted kappa coefficient value of 0.659.

The stomatognathic system was examined using the following instruments: tongue depressors, dental mirror (sterilized), spotlight and front chair for onsite examination. The examiner wore standard personal protective equipment (gloves, cap, mask and lab coat). Evaluation followed a predetermined sequence: palatal evaluation was performed by clinical inspection ,with the examiner making notes about this evaluation sorting the palate between normal or atresic, in sequence, the intercanine distance was measured from the cusp tip, or the center facet of wear, of the canine on one side of the arch to the opposite canine with the help of a digital caliper as established by Moorees in 1966 [9]. Since this methodology an eligible parameter for evaluation of facial cresciento and changes of malocclusion. The distance between the anterior palatine papilla and the limit of the hard palate was obtained with the help of a wooden spatula and measured with a digital caliper. Hard tissue values were assigned to each of the options in the questionnaire: 0 (without alterations), 1 (amelogenesis or dentinogenesis imperfecta) 2 (hypoplasia), 3 (fluorosis) and 4 (tetracycline stains) based on their macroscopic appearance. Setting changes and conceptualization followed the Modified Index of Development Enamel Defects (FDI −1992).

We evaluated the dentofacial alterations of occlusion in deciduous dentition using the standard proposed in the Project SB Brazil 2003 [3], where 0 (no alteration) is attributed to cross compatibility between dental arches (lower arch fully included in above); 1 (crowding) for the misalignment in any of the dental arches or both; 2 (crossbite) when the buccal cusps of the upper posterior teeth are occluding the inferior posterior teeth juices; and 3 (top-to-top) ratio incisors without overbite and overjet are positive with touch between the incisal edges; 4 (open bite) in the absence of touch sagittal canines (canine top cusp tip, occluding the niche between the canine and the first lower molar. Muscle aspects such as: lip posture (presence/absence of contact between upper and lower lip during resting state); resting place of the tongue (between arcs, in the upper or lower arch); breathing pattern (predominantly nasal or oral); depth of the palate (normal or ogival); shape of the upper arch (atretic or semicircular) were auxiliary parameters in defining the diagnosis.

The number of dentofacial changes was established as: 0 (no alterations), 1 (anadontia) no tooth, no evidence of tooth yet to break or early loss; 2 (Supernumerary, number higher than expected), 3 (Twin pregnancy) used for clinical definition of a merger or twinning dental in the absence of radiographic examination complementary for diagnosis.

### The explanatory variables

Explanatory variables, such as the child’s head circumference at birth, the one-minute Apgar score and gestational age, were collected at the moment of birth through the completion of standardized questionnaires. The data for the mother and the newborn of the 2005 cohort were retrieved from the databases.

### Statistics

Profile frequencies and socio-demographic variables were estimated and calculations of prevalence were carried out for each explanatory variable. Associations of categorical variables were assessed using the chi-squared test between the study group and the control groups. Multiple logistic regression was performed considering an explanatory variable, adjusted to a model for the response variable taking into account more than one explanatory variable. (The parametric assumptions were obtained through an ANOVA test and a Kruskal-Wallis test. A statistical significance level of 0.05 was used. EPIINFO 200 was used to build the database and to analyse the data.

### Ethical aspects

The reasons for this study and the methodological procedures involved were explained to the parents and to the school officials. The parents signed a consent form authorizing the participation of their children in this study in accordance with the standards established for research involving human subjects of the National Health Council (Resolution 196/1996 and complementary). The right to leave this study at any time, access to the results and complete confidentiality were all guaranteed. This study fulfills all ethical principles required and was approved by the Ethics Committee of the Universidade Federal de Sergipe (Case 138/2004).

## Results

During the study period, we performed 413 examinations of the stomatognathic system. The two groups had similar distributions for birth weight, type of birth and gender. We evaluated 32 premature children (7.7%) and 381 at term (Table 1). There were no significant differences in maternal age, type of birth, family income, receipt of government assistance, mother’s education, alcoholism and Apgar scores for the first minute, regarding the outcome of the gestational period. Table 2 presents a similar frequency of 47.7% for the term group and 56.2% for the pre-term group, including deleterious habits such as the use of pacifiers and finger sucking habit. Logistic regression showed no statistically significant (p = 0.7679) association between the gestational time, breastfeeding malocclusion, alteration of palate, head circumference, bottle use and parafunctional habits.

**Table 1:** **Socio-demographic characteristics of five-year-old children by gestational term (Aracaju, 2010)**

**Variable**	**Premature**	**Term**	
	**n%**	**n%**	**n%**
**Sex**			
Male	20(62.5)	212(55.6)	232(56.2)
Female	12(37.5)	169(44.4)	181(43.8)
**Hospitalization Category**			
SUS^*^	28(87.5)	329(86.4)	357(86.4)
Health Insurance	4(12.5)	45(11.8)	49(11.9)
Private		6(1.6)	6(1.5)
Missing		1(0.2)	1(0.2)
**Delivery Type**			
Normal	21(65.6)	278(73.0)	299(72.4)
Cesarea	11(34.4)	102(26.8)	113(27.4)
Forceps	1(0.3)		1(0.2)
**Government Aid**			
Unassisted	8(25.0)	139(36.9)	147(35.9)
Assisted	24(75.0)	235(62.3)	259(63.4)
Missing		3(0.8)	3(0.7)
**Familiar Income**			
≤1 MS^ª^	19(59.3)	165(43.3)	184(44.5)
1 a ≤ 2 MS	9(28.2)	124(32.5)	133(32.2)
>2 MS	4(12.5)	72(18.9)	76(18.4)
Missing			20(4.84)
**People living at home**			
2	1(3.1)	27(7.1)	28(6.8)
3 a 4	12(37.5)	186(48.8)	198(48.0)
5 a 8	15(47.0)	142(37.2)	157(37.9)
>8	4(12.6)	26(6.8)	30(5.0)
**Alcohol use**			
Yes	6(18.8)	86(49.4)	92(22.3)
Not	26(81.2)	295(77.4)	32(77.7)
**Mother School level**			
Incomplete Fundamental	16(50.0)	188(49.4)	204(49.3)
Complete Fundamental	13(40.6)	138(36.2)	151(36.6)
Higher education	3(9.4)	46(12.0)	49(11.8)
Missing		9(2.4)	9(2.3)
**Children School type**			
Public	30(97.5)	322(84.5)	352(85.3)
Private	2(6.25)	59(15.5)	61(14.7)

*SUS- Sistema Único de Saúde (Oficial Health System); MS^ª^ Minimum Salary.

**Table 2:** **Clinical Variables of five-year-old children by gestational term (Aracaju, 2010)**

**Variable**	**Premature**	**Term**		
	**n %**	**n%**	***p***	**OR (CI 95%)**
**Mechanical Ventilation**				
Yes	5(15.6)	19(4.9)		
Not	26(81.3)	349(91.7)	0.01	3.53(1.06-11.14)
Missing	1(3.1)	13(3.4)		
**Breastfeeding**				
Yes	27(84.4)	346(90.8)	0.23	1.83(0.58-5.42)
Not	8(15.6)	35(9.2)		
**Bottle use**				
Yes	24(75.0)	264(69.4)		
Not	8(25.0)	110(28.8)	0.59	0.80(0.32-1.94)
Missing		7(1.8)		
**Parafunctional Habits**				
Absent	14(43.7)	199(52.2)		
Pacifier < 1 year	5(15.6)	46(12.0)		
Pacifier > 1 year	11(34.4)	127(33.4)	0.35	1.41(0.64-3,09)
Sucking Finger < 1 year		3(0.8)		
Sucking Finger > 1 year	2(6.3)	6(1.6)		
**Apgar 1st Minute**				
≤3 Changed		3(0.8)	0.61	0.00(0.00-28.50)
3 ≤ 10 Normal	31(96.8)	376(98.7)		
Missing	1(3.2)	2(0.5)		
**Cefalic Perimeter**				
Normal	24(75.0)	356(93.4)		
Changed	6(18.7)	15(3.9)	<0.01	5.93(1.86-18.32)
Missing	2(6.3)	10(2.7)		
**Visit to Dentist**				
Yes	20(62.5)	179(47.0)		
Not	12(37.5)	199(52.2)	0.09	0.54(0.24-1.20)
Missing		3(0.8)		
**Reason to Visit Dentist**				
Not Visited	12(37.4)	199(52.2)		
Periodic	7(21.9)	91(23.9)		
Caries	7(21.9)	45(11.9)	0.17	1.92(0.67-5.62)
Pain	3(9.4)	17(4.5)		
Other	3(9.4)	26(7.6)		

*p =* p-value OR = odds ratio CI = confidence interval.

A total of 21 (5.08%) children had an abnormal head circumference. Premature children were more likely to have a head circumference outside of normal parameters (OR 3.53, 95% CI: 1,06-11,14) and required more mechanical ventilation (OR 5.93, 95% CI: 1.86-18,32), as shown in Table 2.

There was no statistically significant difference with regard to malocclusion between the two groups (Table 3).

**Table 3:** **Changes of malocclusion of five-year-old children at 5 years of age by gestational term (Aracaju, 2010)**

**Variable**	**Premature**	**Term**	
	**n =32**	**n = 381**	
**Malocclusion**	**(%)**	**(%)**	***p***
Normal	14 (43.8)	215(56.3)	
Crowding	2 (6.2)	2 (0.6)	0.056
Crossbite	7 (21.8)	55 (14.5)	0.162
Top to Top	4 (12.5)	56 (14.7)	0.847
Open Bite	5 (15.7)	53 (13.9)	0.492

**Figure 2:**
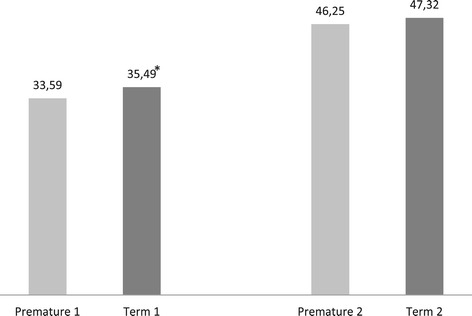
**Intercanine distance and papilla previous limit of the hard palate in 5 years old children according to gestacional term, Aracaju, 2010.** 1 = Medium intercanine; 2 = Medium distance between the papilla and the anterior edge of the hard palate p-Value; 1* = 0,0047; 2 = 0,2368.

**Figure 3:**
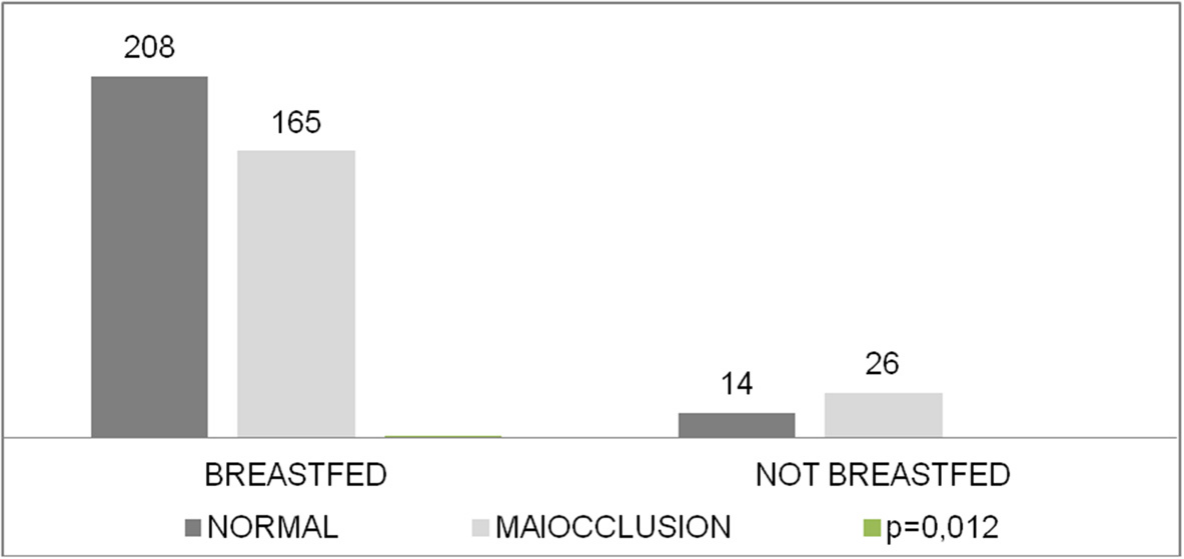
**Dental occlusion in 5 years old children according to breastfeeding, Aracaju, 2010.**

The averege intercanine distance in the group of preterm infants was 33.59 mm, while in term infants it was 35.49 mm (p = 0.004) as shown in Figure 2. A total of 40 (9.68%) children in this study were not breastfed (Figure 3), and of those 26 (65.0%) had some type of malocclusion, showing a significant association between lack of breastfeeding and the development of the stomatognathic system (p = 0.012).

## Discussion

Anomalies in the development of stomatognathic system are associated with premature birth. Unfavorable conditions of preterm birth seem to contribute directly to the occurrence of atresia of the palate and malocclusion.

We found that 75% of preterm infants was bottlefed at an early age, which could be a contributing factor to the high prevalence of malocclusion. Our results showed that 56.2% of this population demonstrated crossbite and 40.6% had palate atresia.

We could not confirm any significant changes in enamel in this population. However, other studies [7-10] have shown that structural changes in enamel hypoplasia in particular can be a relevant factor in preterm groups. However, this study does confirm that the malformation of the palate, predominantly atresia, is a significant and important finding in this population.

The prevalence of prematurity (7.7%) in the studied population is consistent with previous findings [11], which reported the number of preterm births in the northeast region as ranging from 3.8% to 10.2%.

The stomatognathic system comprises static and dynamic oral structures that play important roles in neurovegetative functions (sucking, chewing, swallowing and breathing), speech and facial expressions. Separately or in combination, anomalies resulting from prematurity may unfavorably affect the facial aesthetics [12,13].

Premature children suckling, which is usually not very vigorous, favors the introduction of nutritive and non-nutritive habits such as bottles, pacifiers and finger causing alterations on the morphology of hard palate, modifications on dental positioning and on tongue movement [14-16]. In this study, 10% of children were not breastfed, and of this group, most had some type of malocclusion, demonstrating a significant correlation between the lack of breastfeeding and alterations within the development of the stomatognathic system.

In this study, we report an association between pregnancy outcome and growth of the palate. We measured the intercanine distance, corroborating the previous report by Palmer [17], which reported that the sucking activity has a direct impact on the development of orofacial muscles and that the flexibility and tenderness of the nipple helps to model the palate, reducing the incidence of atresia and malocclusion.

The importance of breast feeding for harmonious growth of the masticatory system is directly related to the proper exercise of the facial muscles in the act of sucking, stimulating muscle tone and development of the temporomandibular joint, providing enough space for the tooth eruption [13]. Our research found a high frequency of bottle feeding, 75% of the preterm group and 69.4% in the term group, but no significant difference between the groups. There was no other breastfeeding technique used and there was not a preventive method to compensate the hypo function of the oral motor system, showing a lack of health education activity in the maternity hospitals of Aracaju.

Learning and refining the techniques required for sucking, swallowing, breathing properly with sucking and hatching and ingesting the correct volume of milk not only increases with gestational age, but also with food experience. The latter occurs late or inefficiently in preterm children, increasing the chance of developmental muscle and skeletal orofacial disorders due to low/ lack of stimulation [18-20].

Structural alterations of skeletal development may also be related to local trauma arising from laryngoscopy or tracheal intubation, which is more common in premature infants [8,21,22]. Atresia is directly related to the malformation caused by the tube. In this study, the preterm infants received significantly more mechanical ventilation. It is noteworthy that at that age, the alveolar bone between the incisal and occlusal surfaces has not yet developed. Therefore, these forces can be severe enough to disrupt and displace the tooth crown and thus lead to higher risk of malocclusion. This disturbance was demonstrated by the preterm group (when compared with the term group), which is in agreement with Seow’s theory [7].

There was a significant association between the head circumference measurement and mechanical ventilation at birth between the two groups, therefore we identified these as risk factors related to premature birth. Epidemiological surveys are useful for highlighting health problems in a population. Through studies like this, it is possible to plan, propose and develop effective treatments for the problems identified here. Therefore, this survey is useful for helping organizations to define health actions that may be required.

The early introduction of artificial feeding, parafunctional habits and oro-tracheal intubation described in this study indeed occur very frequently. Regrettably, public health policies for preterm birth and its consequences are still far from the desired level and more actions are required to encourage improvements and an enhanced understanding of the disease/health process.

## Conclusions

The rates of change of tooth enamel are higher in preterm infants. Changes in head circumference, corrected gestational age, have effective relationship on dental malocclusion. Unfavorable conditions such as mechanical ventilation at birth directly contribute to an increased risk of developmental disorders of the stomatognathic system, the main change being found Enamel hypoplasia. Thus, it is necessary to develop preventive treatments for premature infants, which allow for the proper growth and development of the stomatognathic system. A first step would be to encourage breastfeeding, not only by the neonatologist but also by all health care staff, and to provide more guidance and advice to mothers and families during their child’s recovery process. This would minimize the chances of adverse consequences in premature births.

## Abbreviations

FDI: Modified index of development enamel defects
RG: Pregnancy outcome
ESPHA: Epidemiological Study-Social Health Perinatal Hospital Births Greater Aracaju
